# Interleukin-1-related cytokines as potential biomarkers in autoinflammatory skin diseases

**DOI:** 10.1186/1546-0096-13-S1-P197

**Published:** 2015-09-28

**Authors:** H Bonnekoh, M Maurer, K Krause

**Affiliations:** 1 Charité - Universitätsmedizin Berlin, Dermatology and Allergy, Berlin, Germany

## Introduction

Urticarial rash is a hallmark symptom of autoinflammatory diseases such as Cryopyrin-associated periodic syndrome (CAPS) and Schnitzler's syndrome (SchS). Clinically, the urticarial rash may not be distinguished from the skin symptoms in chronic urticaria patients. As interleukin-1ß (IL-1ß) has been shown to play a pivotal role in the pathogenesis of CAPS and SchS we here aim at investigating IL-1ß and related cytokines for their potential as diagnostic skin biomarkers in patients with urticarial autoinflammatory syndromes.

## Materials and methods

Immunohistochemical stainings (neutrophil marker myeloperoxidase (MPO), IL-1ß, IL-6, IL-18) from lesional skin of patients with CAPS (n=3), SchS (n=9) and chronic spontaneous urticaria (csU) (n=10) as well as healthy control skin samples (n=10) were analyzed by quantitative histomorphometry and compared with cytokine protein concentrations assessed by ELISA.

## Results

Quantitative histomorphometry revealed a higher percentage of neutrophil-dominated dermal cell infiltrate in autoinflammatory diseases that was significant for SchS skin samples as compared with csU samples and healthy controls (p ≤ 0.05). Analysis of IL-1ß, IL-6 and IL-18 positive cells in CAPS and SchS skin showed higher cell numbers which were much less pronounced in csU and healthy control samples. In addition, protein concentrations of all three cytokines were significantly higher in autoinflammatory diseases as compared with csU patients and healthy controls (p ≤ 0.05).

## Conclusion

Our study confirms the predominance of neutrophil-dominated cell infiltrates and demonstrates an upregulation of IL-1-related cytokines in the skin of urticarial autoinflammatory diseases. We suggest to further explore these cytokines as diagnostic biomarkers in larger patient samples.

